# Unraveling the Inflammatory Enigma: Is Neuroinflammation a Culprit or Consequence in Pharmacoresistant Epilepsy?

**DOI:** 10.1002/brb3.71657

**Published:** 2026-08-17

**Authors:** Xianmei Wang, Jie Deng, Dai Shi, Zhiyan Liu, Likun Wang, Guofeng Wu

**Affiliations:** ^1^ Emergency Department the Affiliated Hospital of Guizhou Medical University Guiyang City Guizhou Province China; ^2^ Key Laboratory of Endemic and Ethnic Diseases of the Ministry of Education Guiyang City Guizhou Province China; ^3^ The Second Affiliated Hospital of Guizhou University of Traditional Chinese Medicine Guiyang Guizhou Province China; ^4^ School of Nursing Guizhou Medical University Guiyang City Guizhou Province China

**Keywords:** **Keywords**: cell death, Epilepsy, neuroinflammation, seizures

## Abstract

**Background:**

Approximately 30% of epilepsy patients develop pharmacoresistant epilepsy (PRE), characterized by persistent seizures refractory to antiseizure medications (ASMs). While neuroinflammation is implicated in both epileptogenesis and treatment failure, its precise temporal contribution remains unclear. This study aimed to delineate the distinct roles of acute versus chronic neuroinflammation in seizure susceptibility and the maintenance of drug resistance.

**Methods:**

Male Sprague‐Dawley rats received acute inflammatory priming via systemic (intraperitoneal) or intracranial (hippocampal) lipopolysaccharide (LPS) administration prior to lithium‐pilocarpine‐induced status epilepticus (SE). Rats were subsequently classified as PRE or pharmacosensitive epilepsy (PSE) based on their response to two sequentially administered ASMs. Outcomes were assessed using video‐EEG monitoring, hippocampal cytokine profiling (IL‐6 and TNF‐α), histopathological analysis of neuronal damage, and peripheral inflammatory markers.

**Results:**

Acute inflammatory priming significantly lowered the threshold for SE induction (*P* < 0.05); however, it did not increase the proportion of PRE (*P* > 0.05). In contrast, established PRE was characterized by sustained hippocampal neuroinflammation, with markedly elevated IL‐6 and TNF‐α levels compared to PSE and control groups, alongside severe neuronal loss.

**Conclusion:**

These findings reveal a temporal dissociation in the roles of neuroinflammation: acute inflammation acts as a potent initiator of epileptogenesis, whereas chronic, sustained inflammation coincides with the drug‐resistant phenotype. This study refines the prevailing view of neuroinflammation from a static pathogenic factor to a dynamic, phase‐dependent modulator, supporting the rationale for stage‐targeted strategies in PRE. Notably, putative mechanisms linking inflammation to resistance, such as impaired efferocytosis, warrant future experimental validation.

## 1 Introduction

Epilepsy is one of the most prevalent and debilitating chronic neurological disorders, affecting approximately 52 million people worldwide (GBD Epilepsy Collaborators 2025). Despite the availability of more than 20 antiseizure medications, 30% of patients develop pharmacoresistant epilepsy (PRE), which is characterized by persistent seizures that fail to respond to conventional pharmacological interventions. This condition represents a therapeutic challenge that not only severely compromises quality of life but also imposes substantial economic burdens on global healthcare systems. There is therefore an urgent need to elucidate the mechanisms underlying drug resistance.

PRE research has progressed beyond traditional neuronal excitability models to explore the intricate role of neuroimmune interactions. A compelling body of evidence now proposes neuroinflammation as a cornerstone of both epileptogenesis and treatment failure. Clinically, patients with PRE exhibit elevated levels of pro‐inflammatory cytokines in cerebrospinal fluid and brain tissue (Chen et al., 2023; Hanin et al., 2023). Experiments in animal models have shown that inflammatory mediators can lower seizure thresholds and compromise blood–brain barrier integrity (Soltani Khaboushan et al., 2022). These observations have given rise to a fundamental question: Is neuroinflammation a primary driver of pharmacoresistance or a secondary consequence of recurrent seizures?

This question remains intensely debated owing to seemingly contradictory evidence. Studies have demonstrated that inflammatory processes can directly alter the excitability of the brain and reduce the efficacy of antiseizure drugs, and the authors therefore theorized that inflammation precedes and promotes epileptogenesis and pharmacoresistance (Sumadewi et al., 2023; Yan et al., 2025). Conversely, other researchers argue that neuroinflammation is amplified as a result of recurrent seizures, creating a vicious cycle wherein inflammation and epilepsy mutually reinforce each other, without neuroinflammation necessarily initiating pharmacoresistance (Yan et al., 2025; Sanz and Garcia‐Gimeno, 2020). This unresolved question complicates the therapeutic management of epilepsy, as it remains uncertain whether anti‐inflammatory strategies would prevent the development of pharmacoresistance or merely alleviate symptoms.

Within this context, the concept of inflammatory priming, where an initial inflammatory insult may predispose the brain to a more severe or refractory disease course, has gained traction. However, a critical gap exists in our understanding of how priming prior to epilepsy onset influences the subsequent development of pharmacoresistance. Most previous research has focused on inflammation occurring during or after epileptogenesis; the role of pre‐existing inflammation remains underexplored. To address this knowledge gap, we used well‐established rat models of systemic and intracranial inflammation induced by lipopolysaccharide (LPS) prior to epilepsy induction to investigate whether such inflammatory priming increases seizure susceptibility and influences the development of pharmacoresistance. Through a multi‐methodological approach combining electrophysiological, molecular, and histopathological analyses, we aimed to: (1) determine the effects of inflammatory preconditioning on epileptogenesis and seizure severity, (2) assess the response to first‐line anti‐seizure medications, and (3) evaluate neuroinflammation and neuronal damage.

The aim of our study is to determine the role of neuroinflammation as a culprit or consequence in PRE, and our findings may inform the development of targeted immunomodulatory therapies for patients with PRE. By delineating the temporal relationship between inflammation and the emergence of pharmacoresistance, this study seeks to provide novel insights into the prevention and treatment of this devastating condition.

## 2 Materials and Methods

### 2.1 Animals and Ethics

Male Sprague‐Dawley rats (6–8 weeks old; 160–180 g) were obtained from the Guizhou Medical University Experimental Animal Center and housed as previously described (Li et al., 2024). All experimental procedures were approved by the Guizhou Medical University Animal Care and Use Committee (approval number 2403449) and conducted in accordance with the International Council for Laboratory Animal Science guidelines for the humane treatment of animals.

### 2.2 Inflammatory Modeling and Experimental Groups

**FIGURE 1:**
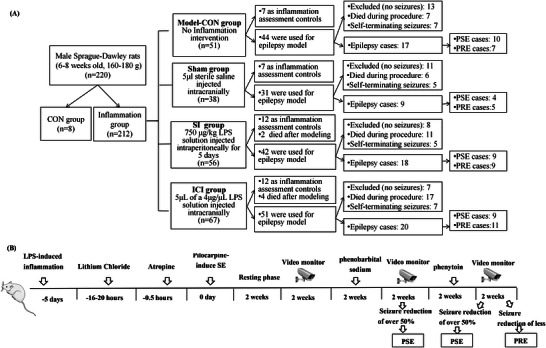
Protocol for generating a pharmacoresistant epilepsy (PRE) rat model and experimental design. (A) Group classification and experimental timeline. (B) Schematic of lithium‑pilocarpine induction with systemic or intracranial LPS priming. Abbreviations: ICI, intracranial inflammation model; Model‐CON, normal control; PRE, pharmacoresistant epilepsy; PSE, pharmacoresponsive epilepsy; Sham, sham operation; SI, systemic inflammation model.

A total of 220 rats were randomly divided into two groups: inflammation model (*n* = 212) and control (CON; *n* = 8). The 212 rats in the inflammation model group were randomly assigned to four experimental groups: intracranial inflammation (ICI; *n* = 67), in which rats received stereotactic hippocampal injections of LPS solution (4 µg/µL, 5 µL) under anesthesia; systemic inflammation (SI; *n* = 56), in which rats received intraperitoneal injections of LPS solution (750 µg/kg) for five consecutive days; sham (*n* = 38), in which rats underwent identical surgical procedures with intracranial injection of 5 µL sterile saline; and normal control (Model‐CON; *n* = 51), in which rats received no interventions. All inflammatory procedures were conducted prior to epilepsy induction, with low mortality observed during this phase (ICI group: 4 rats; SI group: 2 rats) (**Figure** **1A**).

### 2.3 Establishment and Characterization of PRE in Rats With Differential Inflammatory Status

A total of 38 rats were randomly selected from the inflammation model group (Model‐CON: 7; Sham: 7; ICI: 12; SI: 12) to determine baseline inflammatory status. Epilepsy was subsequently induced in the remaining 168 rats (Model‐CON: 44; Sham: 31; ICI: 51; SI: 42) using the lithium chloride‐pilocarpine protocol. Specifically, rats received an intraperitoneal (i.p.) injection of lithium chloride (127 mg/kg; Aladdin, Shanghai, China), followed 18 h later by an i.p. injection of atropine (1 mg/kg; Aladdin, Shanghai, China) to mitigate peripheral cholinergic effects. After a further 30 min, SE was triggered by an initial i.p. injection of pilocarpine hydrochloride (10 mg/kg; Sigma‐Aldrich, St. Louis, MO, USA). Seizure severity was assessed using a modified Racine scale, with SE defined as recurrent seizures of stage 4 or higher lasting at least 30 min. To optimize the induction rate while minimizing mortality, the pilocarpine injection (10 mg/kg, i.p.) was repeated at 30 min intervals for a maximum of 4 times. Diazepam (10 mg/kg; Beijing Yimin Pharmaceutical Co., Ltd., Beijing, China) was administered intraperitoneally 60 min after SE onset to terminate seizures. Rats that did not exhibit SE after the fourth pilocarpine dose (total cumulative dose: 40 mg/kg) were excluded from the study (Model‐CON: 13; Sham: 11; ICI: 7; SI: 8). Additionally, 41 rats died during the acute phase (Model‐CON: 7; Sham: 6; ICI: 17; SI: 11). Video monitoring was initiated 14 days post‐SE and continued for two weeks to identify spontaneous recurrent seizures (SRS). A total of 24 rats without detectable SRS during this period were also excluded (Model‐CON: 7; Sham: 5; ICI: 7; SI: 5).

The screening protocol for drug resistance is detailed in **Figure** **1B**. Initially, 64 epileptic rats were injected intraperitoneally with phenobarbital sodium for two weeks (25 mg/kg as a loading dose, followed by 15 mg/kg twice daily). Seizure reduction was evaluated by comparing the frequency of spontaneous recurrent seizures during a two‐week video monitoring period to the baseline number from the preceding two weeks. Of the 64 rats treated with phenobarbital sodium, 16 exhibited a >50% reduction in spontaneous recurrent seizures and were therefore classified as having pharmacosensitive epilepsy (PSE). The remaining 48 rats, which did not respond to phenobarbital sodium treatment, were subsequently injected intraperitoneally with phenytoin for two weeks (75 mg/kg as a loading dose, followed by 50 mg/kg twice daily). Again, efficacy was assessed using video monitoring. Of the 48 rats treated with phenytoin, 16 exhibited a >50% reduction in spontaneous recurrent seizures and were classified as having PSE. The remaining 32 rats that did not respond to either antiseizure medication were classified as having PRE.

### 2.4 Molecular Analyses (Western Blot and ELISA) and Histopathological Analyses

For western blotting, hippocampal tissues were collected from six rats per group. Proteins were extracted, separated, transferred to membranes, and incubated with primary antibodies recognizing interleukin (IL)‐6 or tumor necrosis factor (TNF)‐α, followed by horseradish peroxidase‐conjugated secondary antibodies. Protein bands were visualized, quantified, and normalized to GAPDH, with each sample tested in triplicate. Analysis was performed blind to prevent bias. Enzyme‐linked immunosorbent assays (ELISAs) were performed using commercial kits to detect IL‐6 and TNF‐α in the hippocampus, with each sample tested in duplicate. Plates were read at a wavelength of 450 nm, and variability was controlled with high and low controls, maintaining an inter‐assay coefficient of variation below 10%. Samples were coded and blinded until after statistical analysis. For histology, brain tissues were fixed, paraffin‐embedded, sectioned, and stained with hematoxylin and eosin or Nissl stain to assess neuronal structure and damage.

### 2.5 Hematological Analyses

Blood samples were collected from the tail veins of inflammation group rats in the 24 h before epilepsy was induced (Model‐CON/Sham: *n* = 7; SI/ICI: *n* = 12). Hematological indices analyzed included the absolute neutrophil count (ANC), neutrophil percentage (NEU%), lymphocyte percentage (LYM%), absolute lymphocyte count (ALC), and total white blood cell count (WBC). The neutrophil‐to‐lymphocyte ratio (NLR) was determined by calculating the quotient of the neutrophil and lymphocyte percentages.

### 2.6 Electroencephalography and Transmission Electron Microscopy

Electroencephalography (EEG) electrodes were implanted into six rats from the CON, PSE, and PRE groups under isoflurane anesthesia. EEG signals were recorded during rest and stimulation using the BL‐420 system (Chengdu TME Technology Co, Ltd., Chengdu, China). Transmission electron microscopy (TEM) was conducted on hippocampal tissues from rats from the same groups, which were fixed, dehydrated, and embedded in epoxy resin. Ultrathin sections were stained and examined under a JEM‐1400FLASH electron microscope (JEOL Ltd., Tokyo, Japan).

### 2.7 Statistical Analysis

Data normality and homogeneity of variance were assessed using SPSS software version 21.0 (IBM Corp., Armonk, NY, USA). Normally distributed data are presented as mean ± standard deviation. Group comparisons were performed using one‐way analysis of variance, with the significance level set at *P* < 0.05. Image analysis was conducted using ImageJ software (National Institutes of Health, Bethesda, MD, USA), and graphs were generated using GraphPad Prism software version 10.2.3 (GraphPad Software, Boston, MA, USA).

## 3 Results

### 3.1 Inflammatory Priming Exacerbates Peripheral Inflammation, Upregulates Central Cytokines, and Increases Susceptibility to Seizures

Hematological analysis revealed that SI and ICI induction significantly altered peripheral immune profiles. Compared with the Sham and Model‐CON groups, the SI and ICI groups exhibited a significantly elevated NEU% (SI vs. Model‐CON, *p* = 0.022; SI vs. Sham, *p* = 0.042; ICI vs. Model‐CON, *P* < 0.001; SI vs. Sham, *P* < 0.001), ANC (SI vs. Model‐CON, *p* = 0.012; SI vs. Sham, *p* = 0.026; ICI vs. Model‐CON, *P* < 0.001; SI vs. Sham, *p* = 0.001), and NLR (SI vs. Model‐CON, *p* = 0.013; SI vs. Sham, *p* = 0.006; ICI vs. Model‐CON, *P* < 0.001; SI vs. Sham, *P* < 0.001), as well as a significantly reduced LYM% (SI vs. Model‐CON, *p* = 0.043; SI vs. Sham, *p* = 0.048; ICI vs. Model‐CON, *p* = 0.004; SI vs. Sham, *p* = 0.008). No significant differences in ALC or WBC were observed among the groups (*P* > 0.05). Pairwise comparisons showed no significant differences between the SI and ICI groups or between the Model‐CON and Sham groups (*P* > 0.05; **Table** **1**). Collectively, these results demonstrate that LPS administration effectively induced a pronounced peripheral inflammatory response.

**TABLE 1:** The hematological inflammation indexes in different inflammatory models.

Variables	Total (*n* = 38)	CON (*n* = 7)	Sham (*n* = 7)	SI (*n* = 12)	ICI (*n* = 12)	*F*	*p*‐value
WBC (×10^−9^/L)	8.5 ± 3.0	6.4 ± 2.7	7.2 ± 2.3	9.3 ± 2.6	9.6 ± 3.4	2.606	0.068
NEU (%)	34.1 ± 13.4	23.2 ± 6.6	24.6 ± 8.4	35.2 ± 8.4^*△^	44.9 ± 14.5^*△^	8.613	< 0.001
LYM (%)	52.4 ± 13.3	61.9 ± 12.7	61.2 ± 13.8	50.1 ± 10.3^*△^	44.5 ± 11.2^*△^	4.479	0.009
NLR	0.7 ± 0.3	0.4 ± 0.1	0.4 ± 0.2	0.7 ± 0.2^*△^	1.1 ± 0.3^*△^	15.882	< 0.001
ANC (×10^−9^/L)	3.0 ± 1.8	1.7 ± 0.6	1.6 ± 1.0	3.4 ± 1.6^*△^	4.3 ± 1.9^*△^	7.575	0.001
ALC(×10^−9^/L)	4.3 ± 1.9	5.0 ± 2.7	3.5 ± 1.3	4.7 ± 1.6	3.9 ± 2.1	0.964	0.421

Abbreviations: ALC, absolute lymphocyte count; ANC, absolute neutrophil count; CON, normal control group; ICI, intracranial inflammation group; LYM, lymphocyte; NEU, neutrophil; NLR, ratio of neutrophil percentage to lymphocyte percentage; SI, systemic inflammation group; WBC, white blood cells.

Group comparisons: “*”denotes *P* < 0.05 vs. CON; “△”denotes *P* < 0.05 vs. Sham.

Statistical analysis: Data are mean ± SD; Statistical analysis was performed using Student's *t*‐test or one‐way ANOVA; *P* < 0.05 was considered significant.

**FIGURE 2:**
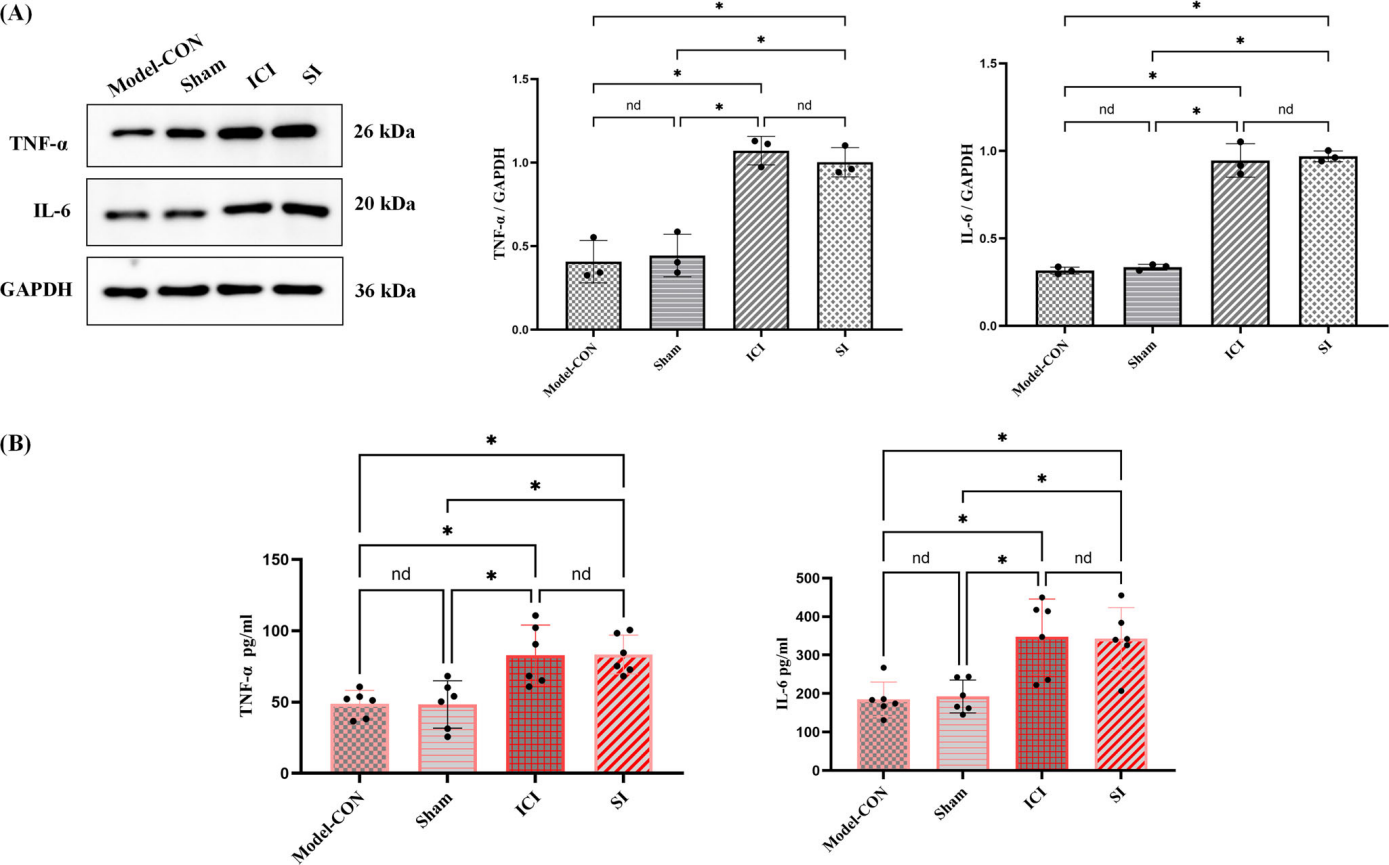
Effects of different treatments on pro‑inflammatory cytokines TNF‑α and IL‑6. (A) Representative Western blots and quantitative analysis. Bar graphs show IL‑6 and TNF‑α levels normalized to GAPDH. (B) ELISA quantification. Data are presented as mean ± SEM; *P* < 0.05; “ns”, not significant (*P* > 0.05).

Rat brain tissues were examined to assess central inflammatory mediators. ELISAs and western blot analyses revealed significantly elevated levels of IL‐6 and TNF‐α in the hippocampus of SI and ICI group rats compared to Sham and Model‐CON group rats (*P* < 0.05; **Figures** **2A** and **2B**). No significant differences were observed between the SI and ICI groups or between the Sham and Model‐CON groups (*P* > 0.05). These results confirm that both SI and ICI significantly upregulate key pro‐inflammatory cytokines in the brain.

Crucially, inflammatory priming markedly increased susceptibility to pilocarpine‐induced SE. The incidence of SE was significantly higher in the ICI (90.5%) and SI (83.3%) groups compared to the Model‐CON (60.8%) and Sham (65.6%) groups (*P* < 0.05). There were no significant differences in SE incidence between the ICI and SI groups or between the Sham and Model‐CON groups (*P* > 0.05; **Table** **2**).

**TABLE 2:** Incidence of epileptic seizures across different experimental groups.

Groups	Total *n* = 200	With *n* = 154	Without *n* = 46	*χ* ^2^	*p*‐value
CON	51	31 (60.8)	20 (39.2)	19.949	< 0.001
Sham	32	21 (65.6)	11 (34.4)		
ICI	63	57 (90.5)^*△^	6 (9.5)^*△^		
SI	54	45 (83.3)^*△^	9 (16.7)^*△^		

Abbreviations: CON, normal control group; ICI, intracranial inflammation group; SI, systemic inflammation group; χ^2^, chi‐square test.

Group comparisons: “*” denotes *P* < 0.05 vs. CON; “△”denotes *P* < 0.05 vs. Sham.

Statistical analysis: Data are *n* (%). Cross‐tabulation was analyzed by chi‐square test; *P* < 0.05 was considered significant.

### 3.2 Inflammatory Priming Does Not Predispose Rats to Pharmacoresistance

**FIGURE 3:**
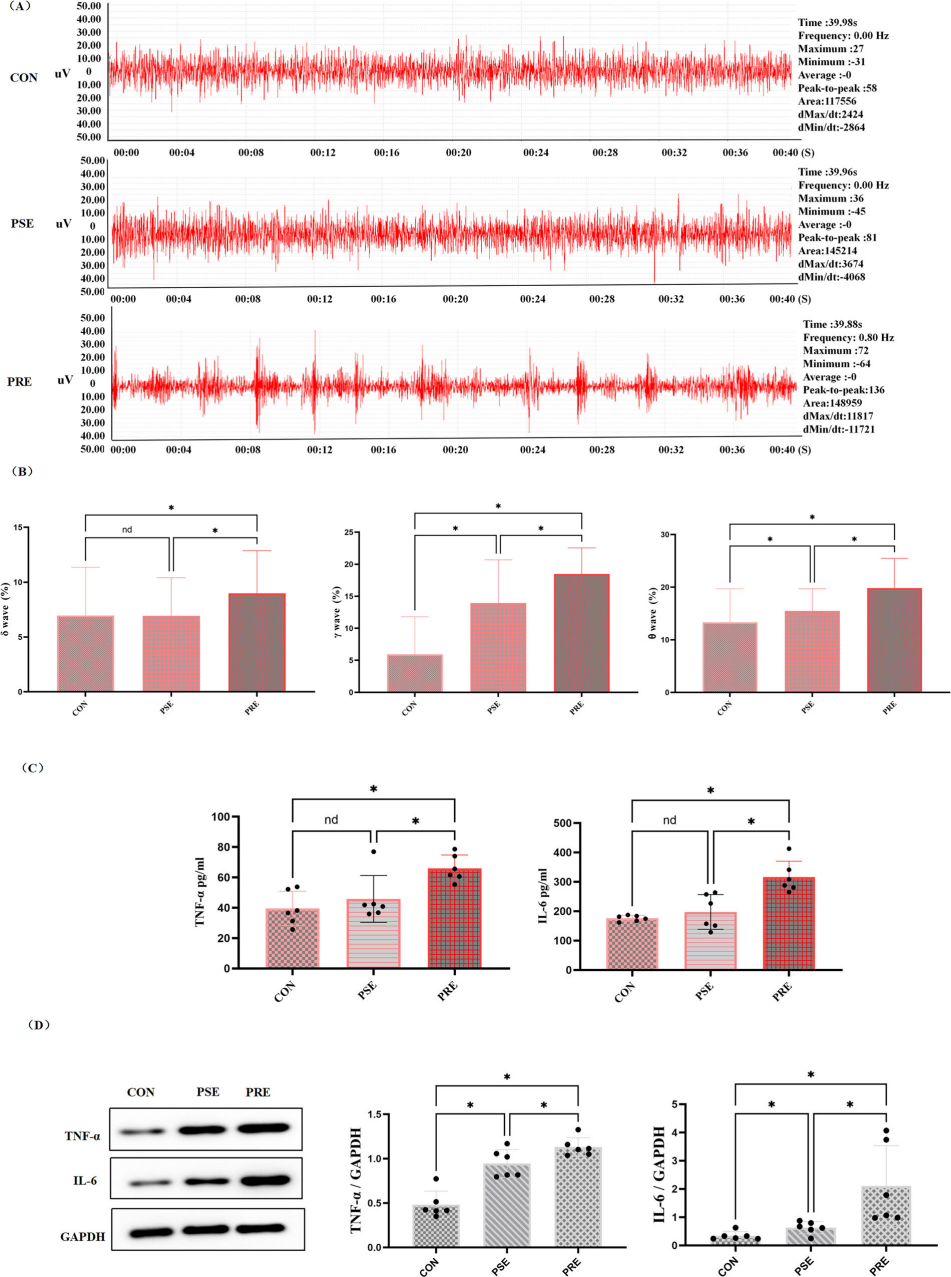
EEG characteristics and inflammatory markers in epileptic rats. (A) Representative EEG traces showing stable activity in controls, increased fluctuations in PSE, and frequent epileptiform spikes in PRE. (B) Power spectral analysis revealed prominent δ, γ, and θ bands in PRE. (C, D) ELISA and Western blot confirmed significantly elevated IL‑6 and TNF‑α in PRE compared with PSE and controls (*P* < 0.05). Data are presented as mean ± SEM; *P* < 0.05; “ns”, not significant.

EEG recordings from rats with PRE consistently showed patterns of abnormal electrical activity, such as spontaneous high‐amplitude spikes, polyspike complexes, and high‐frequency oscillations. These features are consistent with a disrupted excitatory–inhibitory balance and align with expected electrophysiological characteristics following successful epilepsy induction in this model (**Figure** **3A**). Spectral analysis further showed a significant increase in the relative power of δ, γ, and θ waves in the PRE group compared to the PSE and CON groups (**Figure** **3B**), indicating a state of cortical hyperexcitability and disrupted rhythmic brain activity in this group. Behavioral monitoring confirmed that rats with PRE experienced frequent and severe seizures, corresponding to Racine stage ≥3, which is consistent with the observed electrographic severity and supports the translational relevance of the model for studying PRE.

Importantly, inflammatory priming, despite enhancing epileptogenesis, did not translate into a higher incidence of pharmacoresistance, as the proportion of rats with PRE was comparable across the four groups (*P* > 0.05; **Table** **3**).

**TABLE 3:** Incidence of drug‐resistant epileptic seizures in response to inflammatory stimulation.

Groups	Total n = 64	PRE		χ^2^	*p*‐value
		With	Without		
		n = 32	n = 32		
CON	17	7 (21.9)	10 (31.3)	0.841	0.84
Sham	9	5 (15.6)	4 (12.5)		
SI	18	9 (28.1)	9 (28.1)		
ICI	20	11 (34.4)	9 (28.1)		

**Abbreviations**: CON,Normal control group; ICI, Intracranial inflammation group; SI,Systemic inflammation group; χ^2^, chi‐square test.

**Statistical Analysis**: Data are n(%). Cross‐tabulation was analyzed by chi‐square test; P< 0.05 was considered significant.

### 3.3 PRE Is Associated With Exacerbated Neuroinflammation

Rats with PRE exhibited a distinct neuroinflammatory profile. ELISAs and western blot analyses consistently demonstrated significantly elevated concentrations of IL‐6 and TNF‐α in the hippocampal tissue of rats with PRE compared to PSE and CON group rats (*P* < 0.05). No significant differences were detected between the PSE and CON groups (**Figures** **3C** and **3D**). This finding indicates that a sustained neuroinflammatory response is associated with pharmacoresistance rather than the initial seizure injury.

### 3.4 Potential Link Between Inflammation and Neuronal Damage in PRE

**FIGURE 4:**
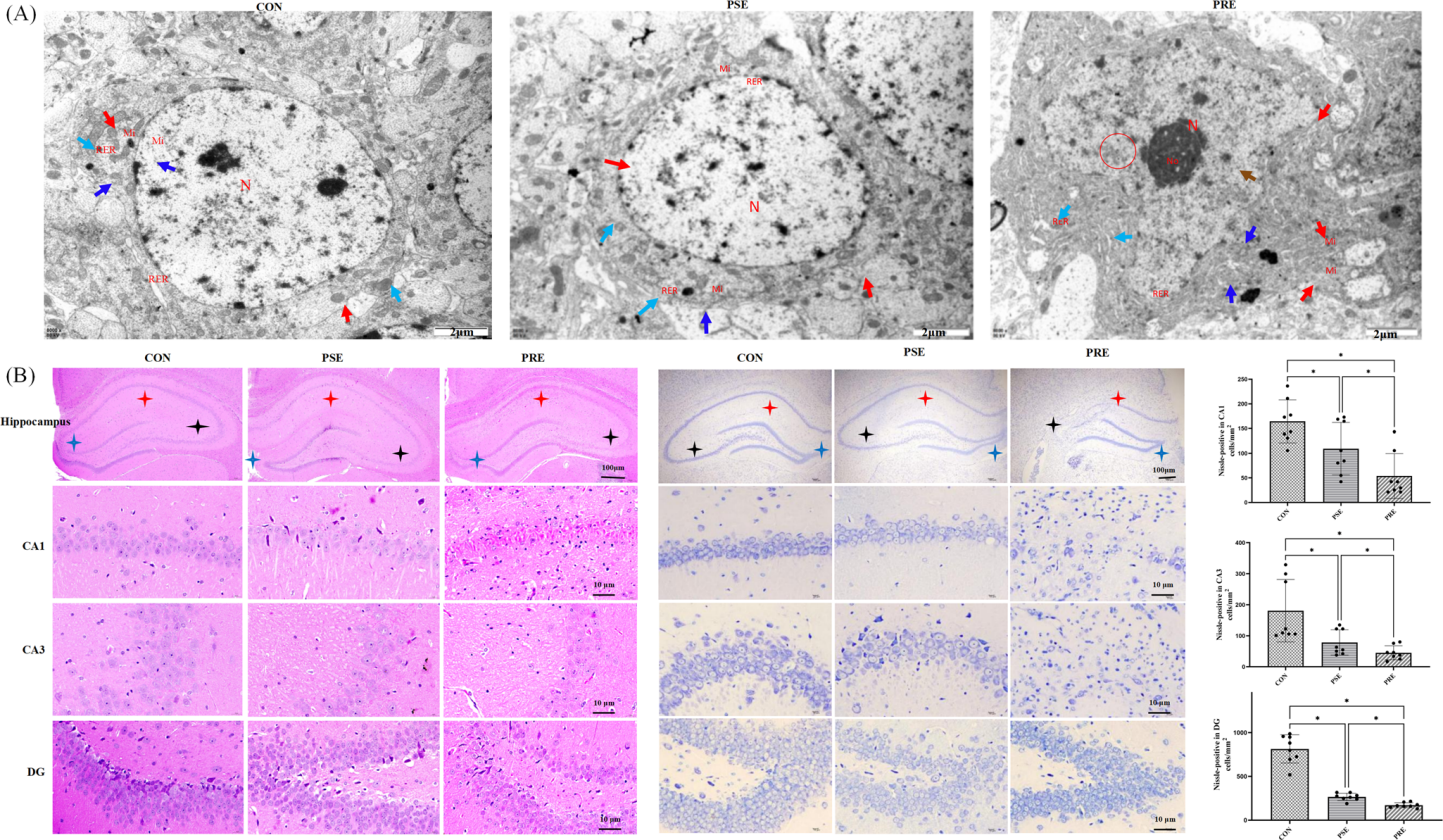
Neuronal damage and cell death across brain regions. (A) Transmission electron microscopy (TEM) showing increased apoptotic cells in PRE. N, nucleus; Mi, mitochondria; RER, rough endoplasmic reticulum. Red arrow, mitochondrion; blue arrow, RER; dark blue arrow, secondary lysosome; deep red arrow, condensed nucleus; red circle, dense chromatin. Scale bar = 2 µm. (B) H&E staining of CA1, CA3, and dentate gyrus (DG). Red, black, and blue crosses indicate representative fields. Scale bar = 20 µm. (C) Nissl staining showing reduced Nissl‑positive neurons in PRE. Scale bar = 20 µm. Data are presented as mean ± SEM; *P*< 0.05;“ ns”, not significant.

Animals with PRE exhibited extensive neuronal damage, as confirmed by both histopathological and ultrastructural analyses. Ultrastructurally, TEM revealed classic features of neuronal apoptosis, including irregular nuclear membranes, mitochondria with swollen morphology and cristae disruption, and severely dilated endoplasmic reticulum (**Figure** **4A**). These cellular‐level injuries corresponded to significant hippocampal tissue damage, with hematoxylin and eosin and Nissl staining showing marked neuronal loss accompanied by gliosis. Quantification of Nissl‐stained neurons revealed a significant reduction in cell density in the PRE group compared to the PSE and CON groups across all examined hippocampal regions (CA1, CA3, and DG; *P* < 0.05; **Figure** **4B**). These findings suggest that severe neuronal damage in PRE may be linked to intense neuroinflammation.

## 4 Discussion

This study explores the potential dual roles of neuroinflammation in epilepsy development and drug resistance. The results reveal that acute inflammation lowers seizure thresholds and amplifies epileptogenesis, yet this transient insult did not predispose rats to pharmacoresistance in our model. In contrast, chronic neuroinflammation emerged as a distinct feature of established PRE, differentiating it from PSE. Rather than defining a strict causal sequence, these findings highlight a temporal dissociation: acute inflammation serves as a potent initiator of epileptogenesis, whereas the maintenance of drug resistance coincides with a sustained neuroinflammatory state. This distinction refines the prevailing view of inflammation in PRE by emphasizing that the duration and persistence of the immune response may be critical determinants of treatment outcomes.

### 4.1 Inflammatory Priming Amplifies Epileptogenesis but Does Not Determine Pharmacoresistance​

#### 4.1.1 Acute Inflammation as a Potent Amplifier of Epileptogenesis

Our data reveal that both systemic and intracranial LPS‐induced inflammatory priming augmented susceptibility to pilocarpine‐induced SE. However, while acute priming increased seizure incidence, it failed to dictate the subsequent development of pharmacoresistance. This observation suggests a potential temporal dissociation between epileptogenesis and drug resistance.

Mechanistically, this heightened susceptibility coincided with indicators of both peripheral and central inflammation, consistent with the concept of a potential systemic‐to‐central interplay. For instance, Sarchi et al. (2024) demonstrated that splenectomy modulates the central inflammatory milieu prior to status epilepticus. However, our observational data do not allow us to infer a directional causal relationship. In our model, robust peripheral recruitment (elevated NLR) occurred concurrently with central inflammatory amplification (elevated hippocampal IL‐6 and TNF‐α). Notably, the analogous effects observed following both systemic and intracranial LPS administration suggest that the presence of central inflammation—regardless of its initiating site—creates a permissive environment for seizures. The observed elevations in central cytokines are compatible with several proposed pathways for lowering seizure thresholds, including enhanced glutamate release (Shim et al., 2018; Ye et al., 2013), reduced GABAergic inhibition (Roseti et al., 2015; Alfano et al., 2022; Zhang et al., 2022; Ahmad et al., 2025), and compromised blood–brain barrier integrity (Gao and Bayraktutan, 2023; Versele et al., 2022). Nevertheless, it remains unclear whether peripheral signals directly initiated the central changes observed or if these changes represent parallel responses to the LPS insult. Collectively, these peripheral and central alterations align with a model wherein immune activation and hyperexcitability are tightly coupled, although further interventional studies are required to disentangle the precise hierarchy of these events (Shi et al., 2024; Beltran‐Velasco and Clemente‐Suárez, 2025).

#### 4.1.2 Acute Inflammatory Priming Alone Is Insufficient to Recapitulate the Pharmacoresistant Phenotype

Critically, inflammatory priming did not increase the proportion of pharmacoresistance in this cohort. While we observed a temporal dissociation between acute priming and later drug resistance, we acknowledge that our study was not designed to exhaustively map the underlying causal mechanisms. Thus, we cannot exclude the possibility that different parameters of acute inflammation (e.g., intensity, repetition) might influence drug responses. Nevertheless, three interrelated considerations may explain why the specific transient insult employed here failed to promote pharmacoresistance. First, acute LPS priming elicited a transient inflammatory surge that subsides well before the assessment of drug responses performed weeks later (Jansen et al., 2023; Shimshek et al., 2025). This limited duration contrasts with the sustained exposure likely required to drive progressive molecular adaptations, such as drug transporter upregulation or network remodeling, which are hallmarks of established PRE (Bazhanova et al., 2021; Weidner et al., 2018). Second, whereas emerging evidence indicates that even brief inflammatory exposures can initiate epigenetic remodeling in glial cells (Sarchi et al., 2024; Tabata et al., 2024; Xiong et al., 2024), the specific molecular signature induced by our acute protocol may not persist sufficiently to alter drug responsiveness, potentially due to compensatory homeostatic mechanisms during the latent period. This distinction emphasizes that not all inflammatory insults are equivalent in their long‐term epigenetic consequences (Wendeln et al., 2018). Third, pharmacoresistance likely emerges through a multi‐hit process (Servilha‐Menezes and Garcia‐Cairasco, 2022). Unlike the brief priming in our model, clinical scenarios and chronic PRE models involve repeated seizures, sustained glial activation, and cumulative structural remodeling over time (Sano et al., 2021; Choy et al., 2022; Kleen et al., 2011). Furthermore, while peripheral LPS can activate central immune signaling via vagus nerve afferents or circumventricular organs, such signaling appears transient and may not establish the enduring glial activation loops characteristic of chronic PRE (Dantzer, 2018; Kim et al., 2024). Thus, within our experimental paradigm, while acute inflammation potently initiates epileptogenesis (as shown in Section 4.1.1), it appears unlikely to be sufficient to sustain the complex, self‐perpetuating drug‐resistant state under our experimental conditions.

#### 4.1.3 Implications for Temporal Windows in Epilepsy Therapeutics

Rather than representing a limitation, the absence of a direct link between acute priming and pharmacoresistance highlights a potential temporal boundary in epilepsy pathophysiology. As suggested in Section 4.1.2, this dissociation implies that the impact of inflammation is likely phase‐specific. While acute, transient inflammation coincided with the initiation of epileptogenesis, the transition to a chronic, self‐perpetuating inflammatory state was a distinguishing feature of established pharmacoresistance (Hoffman et al., 2026). This distinction refines the prevailing conceptual framework in two ways. First, it underscores that not all inflammatory insults are equivalent; the nature, duration, and cellular profile (e.g., brief cytokine spikes vs. prolonged glial activation) appear to dictate pathological outcomes (Zawadzka et al., 2025; Onat et al., 2025). Second, it generates a testable hypothesis regarding therapeutic timing. Our data suggest that anti‐inflammatory interventions might be optimally timed to specific disease phases: targeting acute inflammation could prevent epilepsy, whereas disrupting chronic inflammation may be necessary to reverse established pharmacoresistance (Yu et al., 2020). By delineating these associations, we move away from a binary view of inflammation toward a nuanced, time‐based model for precision medicine in PRE.

### 4.2 Chronic Neuroinflammation in Established PRE: A Persistent Feature Associated With Drug Resistance

In contrast to the transient nature of acute priming, established PRE was characterized by persistent and accentuated neuroinflammation, as evidenced by markedly elevated hippocampal IL‐6 and TNF‐α levels. Crucially, while this sustained inflammation emerged as a distinguishing feature between PRE and PSE, our observational data preclude definitive causal inference. This finding aligns with the concept that chronic neuroinflammation may contribute to the pharmacoresistant phenotype through complex, multifactorial interactions, rather than acting as its sole determinant (Bazhanova et al., 2021). Based on existing literature, several potential contributory pathways exist. Although we did not directly measure IL‐1β or glial markers, previous reports indicate that pro‐inflammatory cytokines (including IL‐1β and TNF‐α) can increase drug efflux via transporters and alter neuronal targets (e.g., GABAA and glutamate receptors), which may compromise drug efficacy (Hartz et al., 2017; Lemes et al., 2025). Additionally, chronic seizures are known to cause reactive gliosis; activated microglia and astrocytes can release cytokines that disrupt glutamate homeostasis and promote hyperexcitability (Wilcox and Vezzani, 2014; Rana and Musto, 2018). In this context, the co‐occurrence of elevated cytokines and drug resistance in our model is consistent with a potential self‐perpetuating cycle: recurrent seizures may trigger or exacerbate inflammation, which in turn could sustain pharmacoresistance, further driving seizures (Sanz and Garcia‐Gimeno, 2020). However, we acknowledge that this relationship is likely bidirectional and modulated by genetic, metabolic, and network‐level factors. Breaking this cycle represents a promising therapeutic strategy. Preclinical studies have demonstrated efficacy of interventions targeting IL‐1 or TNF‐α (Zhang et al., 2022; Yamanaka et al., 2021). Aligning with our temporal model (Section 4.1.3), therapeutic timing appears critical. Such therapies may be most effective during the transition to chronicity; once structural and network remodeling become entrenched, reversing pharmacoresistance may prove considerably more challenging. Therefore, future clinical trials should stratify patients by inflammatory status to rigorously test these immunomodulatory approaches.

### 4.3 Potential Link Between Neuronal Damage and Inflammation

Our data reveal a positive association between severe neuronal loss and elevated hippocampal cytokines in PRE. While we did not directly assess specific cell death modalities or clearance mechanisms, this observation aligns with existing literature suggesting that the release of damage‐associated molecular patterns (DAMPs) from dying neurons can trigger innate immune responses (Feng et al., 2024; Li et al., 2024). Extending from this, impaired clearance of cellular debris (efferocytosis) by glial cells has been proposed as a potential mechanism that may prolong this pro‐inflammatory state (Doran et al., 2020; Mehrotra and Ravichandran, 2022). Although these pathways were not mechanistically interrogated in the present study, the putative link between neuronal damage and unresolved inflammation warrants further investigation. If validated, strategies aimed at enhancing the resolution of inflammation (e.g., improving efferocytosis) could represent a novel therapeutic avenue for PRE.

### 4.4 Limitations and Future Directions

Several limitations constrain interpretation of our findings. First, we restricted analyses to bulk hippocampal IL‐6/TNF‐α without glial markers (Iba‐1/GFAP) or BBB integrity assessments, so we cannot distinguish central cytokine synthesis from peripheral infiltration/leakage. Omitting IL‐1β also limited inflammatory profiling breadth. Second, our two‐drug failure PRE criterion aligns with preclinical standards but differs from the ILAE clinical definition requiring adequately trialed/tolerated anti‐seizure medications, warranting caution when extrapolating to humans. Third, proposed links between efferocytosis, epigenetic remodeling, and pharmacoresistance are strictly hypothesis‐generating, as we did not directly assay these processes. Finally, high animal exclusion during SE induction may introduce selection bias, potentially limiting generalizability (Figure 1). Future studies should expand cytokine profiling (including IL‐1β), integrate single‐cell transcriptomics to resolve cell‐specific mechanisms, and directly validate efferocytosis/epigenetic hypotheses via targeted in vivo assays. Prospective testing of phase‐specific anti‐inflammatory therapies will be critical to verify our proposed therapeutic window. Ultimately, identifying peripheral/CSF inflammatory biomarkers for patient stratification will advance precision medicine for PRE.

## 5 Conclusion

This study reveals a temporal dissociation in the roles of neuroinflammation in epilepsy. While acute inflammation lowered the threshold for epileptogenesis, it did not predispose rats to pharmacoresistance, likely reflecting its transient nature. In contrast, established PRE was characterized by chronic neuroinflammation, coinciding with​ a drug‐resistant phenotype potentially driven by progressive molecular and network alterations. Notably, putative mechanisms such as impaired efferocytosis warrant future validation. These findings refine the prevailing view of inflammation from a static pathogenic factor to a dynamic, phase‐dependent modulator. Consequently, they underscore the importance of stage‐specific anti‐inflammatory strategies, providing a rationale for precision medicine approaches targeting chronic inflammation in PRE.

## Author Contributions

**Xianmei Wang**: visualization, project administration, methodology, funding acquisition, data curation, Writing – review and editing. **Jie Deng**: formal analysis, methodology. **Dai Shi**: validation, software, resources. **Zhiyan Liu**: funding acquisition, supervision, writing – review and editing, visualization. **Guofeng Wu**: funding acquisition, resources, writing – original draft. **Likun Wang**: funding acquisition, resources, supervision.

## Funding

This research received support from the Research Start‐up Fund Project for High‐level Talents of Guizhou Medical University (J [2025] 34 (University‐Doctoral Program Collaboration Series)), the Teaching Research Funding Project of the Affiliated Hospital of Guizhou Medical University (School of Clinical Medicine) (JXYJY‐2025‐062), the General Program of the 2026 Provincial Basic Research Plan (No. QKH‐JC‐MS [2026]520), the National Natural Science Foundation Cultivation Program of Guizhou Medical University (2026NSFCP14), the 2025 Annual Scientific Research Project of Guizhou Provincial Nursing Association (GZHLKY202507), the Natural Science Foundation of China (82460272), the Leading Discipline Program of the Affiliated Hospital of Guizhou Medical University (gyfyxkrc‐2023–05), the Key Lab of Acute Brain Injury and Function Repair in Guizhou Medical University [2024]fy007, the Key Advantageous Discipline Construction Project of Guizhou Provincial Health Commission in 2023 in Emergency Department, and the Guizhou Province Clinical Research Center for Emergency Medicine (No. LCZX[2025]004).

## Consent

All authors gave their consent for publication.

## Conflicts of Interest

The authors declare no conflicts of interest.

## Declaration

The study's animal protocols were approved by the local academic and ethics committee (approval number 2403449).

## Data Availability

All data generated or analyzed during this study are included in this published article.
